# A pilot study: Metabolic profiling of plasma and saliva samples from newly diagnosed glioblastoma patients

**DOI:** 10.1002/cam4.5857

**Published:** 2023-04-09

**Authors:** Juliana Muller Bark, Avinash V. Karpe, James D. Doecke, Paul Leo, Rosalind L. Jeffree, Benjamin Chua, Bryan W. Day, David J. Beale, Chamindie Punyadeera

**Affiliations:** ^1^ Faculty of Health, Centre for Biomedical Technologies School of Biomedical Sciences, Queensland University of Technology Brisbane Queensland Australia; ^2^ Saliva and Liquid Biopsy Translational Laboratory Griffith Institute for Drug Discovery – Griffith University Brisbane Queensland Australia; ^3^ Faculty of Health School of Biomedical Sciences, Queensland University of Technology Gardens Point Queensland Australia; ^4^ Environment, Commonwealth Scientific and Industrial Research Organization (CSIRO), Ecosciences Precinct Dutton Park Queensland Australia; ^5^ Agriculture and Food Commonwealth Scientific and Industrial Research Organization (CSIRO) Acton Australian Capital Territory Australia; ^6^ Australian eHealth Research Centre, CSIRO. Level 7, Surgical Treatment and Rehabilitation Service – STARS Royal Brisbane and Women's Hospital Herston Queensland Australia; ^7^ Faculty of Health, Translational Genomics Group School of Biomedical Sciences, Queensland University of Technology Woolloongabba Australia; ^8^ QIMR Berghofer Medical Research Institute Herston Queensland Australia; ^9^ Faculty of Medicine University of Queensland Herston Queensland Australia; ^10^ Kenneth G. Jamieson Department of Neurosurgery Royal Brisbane and Women's Hospital Brisbane Queensland Australia; ^11^ Cell and Molecular Biology Department, Sid Faithfull Brain Cancer Laboratory QIMR Berghofer MRI Brisbane Queensland Australia; ^12^ Cancer Care Services Royal Brisbane and Women's Hospital Brisbane Queensland Australia; ^13^ Menzies Health Institute, Griffith University Southport Queensland Australia; ^14^ Translational Research Institute Woolloongabba Queensland Australia

**Keywords:** blood, glioblastoma, lipids, metabolites, metabolomics, saliva

## Abstract

**Background:**

Despite aggressive treatment, more than 90% of glioblastoma (GBM) patients experience recurrences. GBM response to therapy is currently assessed by imaging techniques and tissue biopsy. However, difficulties with these methods may cause misinterpretation of treatment outcomes. Currently, no validated therapy response biomarkers are available for monitoring GBM progression. Metabolomics holds potential as a complementary tool to improve the interpretation of therapy responses to help in clinical interventions for GBM patients.

**Methods:**

Saliva and blood from GBM patients were collected pre and postoperatively. Patients were stratified conforming their progression‐free survival (PFS) into favourable or unfavourable clinical outcomes (>9 months or PFS ≤ 9 months, respectively). Analysis of saliva (whole‐mouth and oral rinse) and plasma samples was conducted utilising LC‐QqQ‐MS and LC‐QTOF‐MS to determine the metabolomic and lipidomic profiles. The data were investigated using univariate and multivariate statistical analyses and graphical LASSO‐based graphic network analyses.

**Results:**

Altogether, 151 metabolites and 197 lipids were detected within all saliva and plasma samples. Among the patients with unfavourable outcomes, metabolites such as cyclic‐AMP, 3‐hydroxy‐kynurenine, dihydroorotate, UDP and cis‐aconitate were elevated, compared to patients with favourable outcomes during pre‐and post‐surgery. These metabolites showed to impact the pentose phosphate and Warburg effect pathways. The lipid profile of patients who experienced unfavourable outcomes revealed a higher heterogeneity in the abundance of lipids and fewer associations between markers in contrast to the favourable outcome group.

**Conclusion:**

Our findings indicate that changes in salivary and plasma metabolites in GBM patients can potentially be employed as less invasive prognostic biomarkers/biomarker panel but validation with larger cohorts is required.

## Graphical abstract

Saliva and plasma samples were collected from 21 newly diagnosed GBM patients pre and post‐operatively. Metabolomic and lipidomic profiles were analysed using LC‐QqQ‐MS and LC‐QTOF‐MS. GBM patients were classified as favourable or unfavourable patients according to clinical outcomes (progression‐free survival (PFS) >nine months or PFS ≤nine months, respectively). The data were investigated using univariate and multivariate statistical analyses and graphical LASSO‐based graphic network analyses.

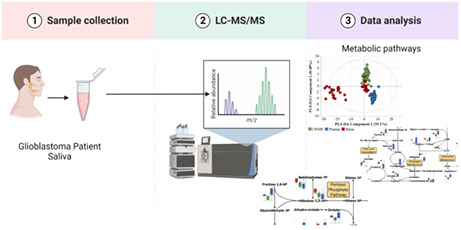

## 1 INTRODUCTION

Glioma remains the most common brain cancer type in adults.^1^ Among gliomas, glioblastoma (GBM)^1^ is the most frequent and deadliest subtype.^2^, ^3^ Patients undergo an aggressive multimodal treatment; nevertheless, nearly all patients suffer from disease recurrence.^4^, ^5^, ^6^, ^7^, ^8^ GBM response to therapy is assessed by imaging techniques and tissue biopsy.^9^ Nevertheless, limitations within these techniques may result in misinterpretation of treatment response and delay clinical interventions.^10^, ^11^ Additionally, tissue biopsies are highly invasive and fail to identify dynamic alterations in the tumour. Currently, no clinically validated maker has been established to monitor GBM progression over the course of treatment.^11^, ^12^ To overcome these limitations, liquid biopsies, i.e., the analysis of tumour biomarkers sampled from body fluids,^13^ have emerged as a potential approach to capture tumour activity non‐invasively.

Metabolic alteration is a cancer cell hallmark,^14^ allowing cells to reprogram their metabolism to continuously support their growth. Metabolomics is defined as the study of small molecules generated by metabolic reactions within a biological sample.^15^ Metabolomics data contain a wealth of information that reflects underlying diseases and has been applied in research for the detection of disease‐specific biomarkers.^16^ These small molecules can be measured in blood, saliva and other body fluids,^15^, ^16^ offering a minimally invasive approach to monitor metabolic changes in biological samples. Despite blood being widely studied in medical settings, saliva is emerging as a viable alternative for biofluid analysis.^17^, ^18^ Collection is simple, affordable and no special equipment or personnel is required. Saliva composition also changes under certain medical conditions, such as cancer, making it a valuable tool for diagnosis and monitoring cancer.^17^, ^19^, ^20^, ^21^, ^22^, ^23^ As a subset of the metabolome, the lipidome can be explored using lipidomics (i.e., analysis of the structure and function of all lipids generated by a particular cell or organism).^15^, ^24^

There is currently limited data relating to metabolomics and lipidomics profile changes in body fluids of glioma patients. However, distinct metabolic profiles have been reported between high and low‐grade brain tumours.^25^, ^26^ Baranovičová et al. identified higher levels of tyrosine and phenylalanine exclusively in plasma of GBM patients compared to other glioma types.^27^ Altered levels of uracil, arginine, lactate, cystamine and ornithine in glioma patients correlated with the isocitrate dehydrogenase (IDH) mutation status.^28^ Also, the differential abundance of arginine, methionine and kynurenate detected in plasma samples was associated with a 2‐year overall survival in GBM patients.^29^ Similarly, alterations in the lipid metabolism of glioma patients have been described.^30^, ^31^, ^32^ Patients with IDH mutation had decreased levels of triglycerides and sphingolipids while membrane phosphatidyl lipids were not altered.^30^, ^31^ Interestingly, Wu et al. described a lipid metabolism‐gene set as a prognostic factor in gliomas.^32^ These findings highlight the importance of metabolomics/lipidomics for identifying specific and sensitive biomarkers to improve the prognostication in GBM in a minimally invasive way. Therefore, this pilot study aims to identify salivary and plasmatic differentially expressed metabolites and lipids in pre and post‐operative GBM patients and explore their potential prognostic value.

## 2 MATERIALS AND METHODS

### 2.1 Ethics approval

This study obtained approval from the human research ethics committees of Royal Brisbane and Women's Hospital (Approval number: HREC/2019/QRBW/48780), Queensland University of Technology (Approval number: 1900000292), and The Griffith University Human Research Ethics Committee (GUHREC Ref No: 2022/061). All patients in this study provided written consent to participate and had blood and saliva samples collected before and after undergoing brain surgery or a needle biopsy. Sample collection was carried out between June 2019 and January 2021.

### 2.2 Patient cohort and sample collections

Newly diagnosed GBM patients were recruited for this study. Blood (*n* = 21) and saliva (*n* = 18) samples were collected before and after surgery (within 2 weeks). Saliva was collected using two different methods, unstimulated whole mouth saliva (‘drool’, UWMS) and oral rinse, as previously described.^33^, ^34^, ^35^, ^36^ Briefly, before saliva collection, patients were requested to fast and to rinse their mouths. For unstimulated saliva, volunteers were asked to seat comfortably with the head slightly tilted forward for about 2–5 min, and then collect their saliva in 50 mL falcon tubes. For oral rinse samples, patients were asked to alternate between swishing and gargling a 0.9 (1)% saline solution for 1–2 min and samples (20 mL) were collected in 50 mL falcon tubes.^37^, ^38^ Samples were put onto ice, aliquoted and stored at −80°C. For plasma samples, whole blood was collected in EDTA tubes by an accredited phlebotomist or medical staff at the hospital. Blood samples were centrifuged at 500 ***g*** for 15 min to obtain plasma. The plasma was aliquoted and stored at −80°C until further analysis.

### 2.3 LC–MS‐based metabolomics

The LC–MS‐based metabolomics analysis was carried out as previously described by Gyawali et al^39^ using the Agilent Metabolomics dMRM Database and Method.^40^ All chemicals were sourced from Merck (Merck Australia). Internal standard isotopes were sourced from Cambridge Isotope Laboratories.

### 2.4 LC–MS‐based lipid profiling

The LC–MS‐based lipid extraction and analysis was performed as previously reported^39^, ^41^ using the Agilent 6546 LC‐QTOF‐MS coupled with an Agilent Infinity II Flex UHPLC system employing the Agilent Metabolomics dMRM Database and Method.^40^

### 2.5 Chemometrics and statistical analysis

Chemometrics and statistical analysis were conducted as previously described by Gyawali et al^39^ and Beale et al,^41^ in line with the guidelines of Metabolomics Standards Initiative {Sumner, 2007 #852}{Spicer, 2017 #853}{Cajka, 2016 #854}. Briefly, univariate and multivariate statistical analyses were performed using SIMCA 16.1 (Umetrics AG) and MetaboAnalyst 5.0. Batch effect correction was done for metabolomics and lipidomics datasets using default single value decomposition (SVD) method, followed by the Least distance analysis within Batch Effect Correction tool of Metaboanalyst 5.0. Metabolomic data were then normalised to internal standards L‐phenylalanine (1‐^13^C) (RSD = 13.34%) and QC mix (1 QC mix per 10 samples; RSD = 1.31%–14.87%, Table S2).

The batch‐corrected chromatographic data were subjected to Log10 transformation and Pareto scaling before univariate and multivariate statistical analysis. Multivariate analyses of metabolomic and lipidomic datasets were done through principal component analysis (PCA), followed by partial least square‐determinant analysis (PLS‐DA). To determine the false discovery rate (FDR) univariate (using one‐way anova) and multivariate (using Significance Analysis of Microarray (SAM)) analyses were performed.

Univariate analysis was performed through *t* test and one‐way anova as per the previously reported study.^42^ The biomarker analysis was performed using Omics skin toolbox of SIMCA 16.1 and Biomarker tool box of Metaboanalyst 5.0.^43^ Biomarker lipids and metabolites were shortlisted and ranked using an area under the ROC curve (AUROC) and T‐statistics at 95% confidence interval as reported previously{Karpe, 2022 #855}. The metabolic pathway networks obtained after statistical analyses were then manually curated in Omix visualisation software (Version 1.9.34; Omix Visualisation GmbH and Co. KG). In addition to the previously described analysis, basic comparisons of biomarker means were computed for lipids across all samples, along with an assessment using a linear mixed effects model (random effects for saliva sample type). Graphical network analyses using the graphical LASSO were performed for favourable/unfavourable patients.

## 3 RESULTS

### 3.1 Patient characteristic

We recruited 21 GBM patients for this observational study. This cohort consists of 10 men and 11 women with a mean age of 62, ranging from 37 to 82 years. A total of 20 patients presented with IDH wild type and one patient with IDH mutant which at the time of collection was classified as GBM according to the WHO Classification of Tumours of the Central Nervous System.^1^ The clinical outcomes of these patients were classified as favourable or unfavourable according to their progression‐free survival (PFS). PFS rates longer or equal to 9 months were considered favourable outcomes, while PFS shorter than 9 months were considered unfavourable (Table S1). PFS data was determined using the amount of time between the sample collection date of preoperative blood or saliva and the time of progression, death or the last follow‐up visit with an MRI scan image. Patients without information available to conclude their outcome or with a time elapsed of <9 months and no evidence of progression were excluded from the prognostic analyses. For plasma samples, out of the 21 patients, 12 presented with unfavourable outcomes, six with favourable and for three the outcome data were not available. Due to COVID‐19, there were restrictions at the hospital; therefore, not all patients had saliva samples collected. For saliva samples, out of 18 patients, 11 were considered to have unfavourable outcomes, 6 with favourable and one patient did not have outcome data available. No information was available regarding the prognosis of the only patient with IDH mutation, and this patient was excluded from the prognosis analysis.

### 3.2 Unique metabolomics and lipidomics profiles for GBM patients' pre‐ and post‐surgery saliva and plasma samples

**FIGURE 1:**
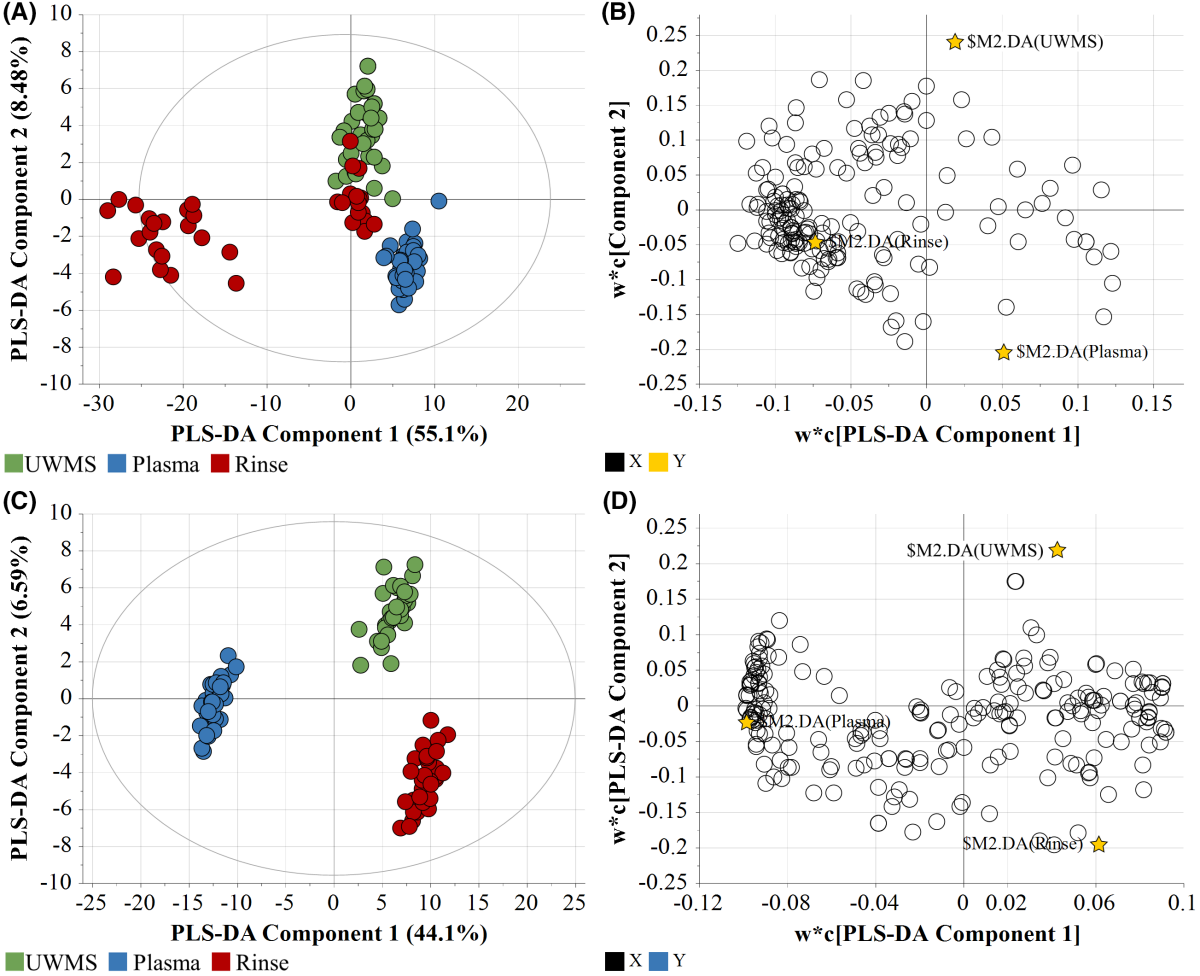
PLS‐DA dataset for the metabolic and lipidomic profiles across all samples of glioblastoma patients. (A) Spread of metabolite samples indicated by score scatter plot, with ellipse representing 95% confidence interval, and (B) Spread of metabolites concerning the groups, indicated by the loading scatter plot. *N* = 125, *R* ^2^ *X* = 0.802; *R* ^2^ *Y* = 0.956; *R* ^2^ = 0.892. (C) Spread of lipid samples indicated by score scatter plot, with ellipse representing 95% confidence interval, and (D) Spread of lipids concerning the groups, indicated by the loading scatter plot. *N* = 125, *R* ^2^ *X* = 0.58; *R* ^2^ *Y* = 0.9825; *R* ^2^ = 0.948.

The LC‐QQQ‐MS analysis identified 161 metabolites across all samples (oral rinse, UWMS and plasma). Of these, Fisher's least significant difference testing via one‐way anova revealed 151 metabolites to be statistically significant (FDR adjusted *p* ≤ 0.05). LC‐QTOF‐MS‐based lipidomic analysis identified 227 lipids across all samples, of which 197 were statistically significant. Given inherent variability in the data set supervised, multivariate regression, partial least squares – discriminant analysis (PLS‐DA) was applied. While differences were seen, some overlap of metabolic profiles between UWMS and rinse samples was observed with the PLS‐DA model (Figure 1A,B).

The OPLS‐DA indicated good linearity (R^2^) and predictability (*Q* ^2^), with good separation between the metabolite dataset (*R* ^2^ *X* = 0.805; *R* ^2^ *Y* = 0.895; *R* ^2^ = 0.93) profiles (Figure S1). The cross‐validation analysis using CV‐anova of metabolomics dataset indicated the model to be statistically significant (regression sum of squares (SS) = 35.62, mean‐squared error (MS) = 8.91 and *p* = 1.13e^−07^, standard deviation (SD) = 2.98).

The PLS‐DA output of lipidomic profile was not as good as that of the metabolites, (*R* ^2^ *X* = 0.58; *R* ^2^ *Y* = 0.0.983; *R* ^2^ = 0.948), it was enough to produce a statistically significant discrimination (Figure 1C,D). When analysed through CV‐anova validation, the lipidomic model showed statistical significance (SS = 118.19, MS = 29.55 and *p* = 2.32e^−36^, SD = 3.47).

**FIGURE 2:**
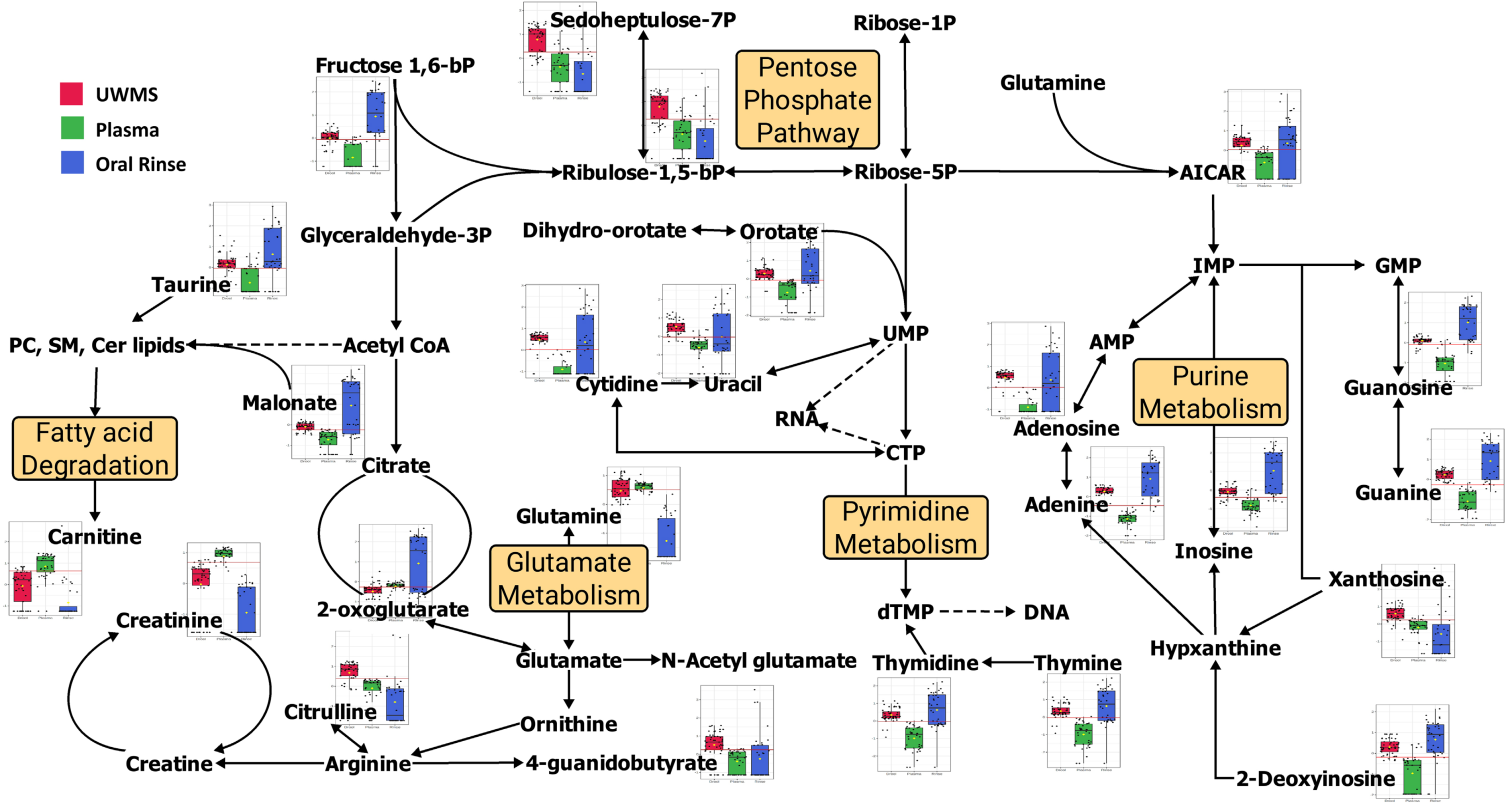
Key metabolites and pathways observed in UWMS, rinse, and plasma samples of glioblastoma patients pre and post‐surgery. The graphs in the figure indicate the Log2Fold Change of the statistically significant metabolites identified (FDR ≤ 0.05; ROAUC ≥ 0.85). AICAR, 5‐aminoimidazole‐4‐carboxamide‐1‐β‐d‐ribofuranoside; AMP, adenosine monophosphate; Cer, ceramides; CTP, cytidine triphosphate; dTMP, thymidylate; DNA, deoxyribonucleic acid; GMP, guanosine monophosphate synthetase; IMP, inosine monophosphate; RNA, ribonucleic acid; PC, phosphatidylcholines; SM, sphingomyelin; UMP, uridine monophosphate.

The 151 statistically significant metabolites were enriched in 49 metabolic pathways. Among these, nucleotide metabolism pathways and non‐aromatic amino acid metabolism were observed to be most represented (Table S2). The univariate analysis indicated enrichment in the nitrogen metabolism in the form of either nucleotide intermediates or amino acid intermediates (Figure S2). In addition, the biochemical profile showed the predominant central carbon metabolites to have a higher level in saliva than in plasma, except for kynurenine (Figure S2) and creatinine. The key metabolites and pathways observed in all salivary and plasmatic samples of patients pre and post‐surgery can be observed in Figure 2.

**FIGURE 3:**
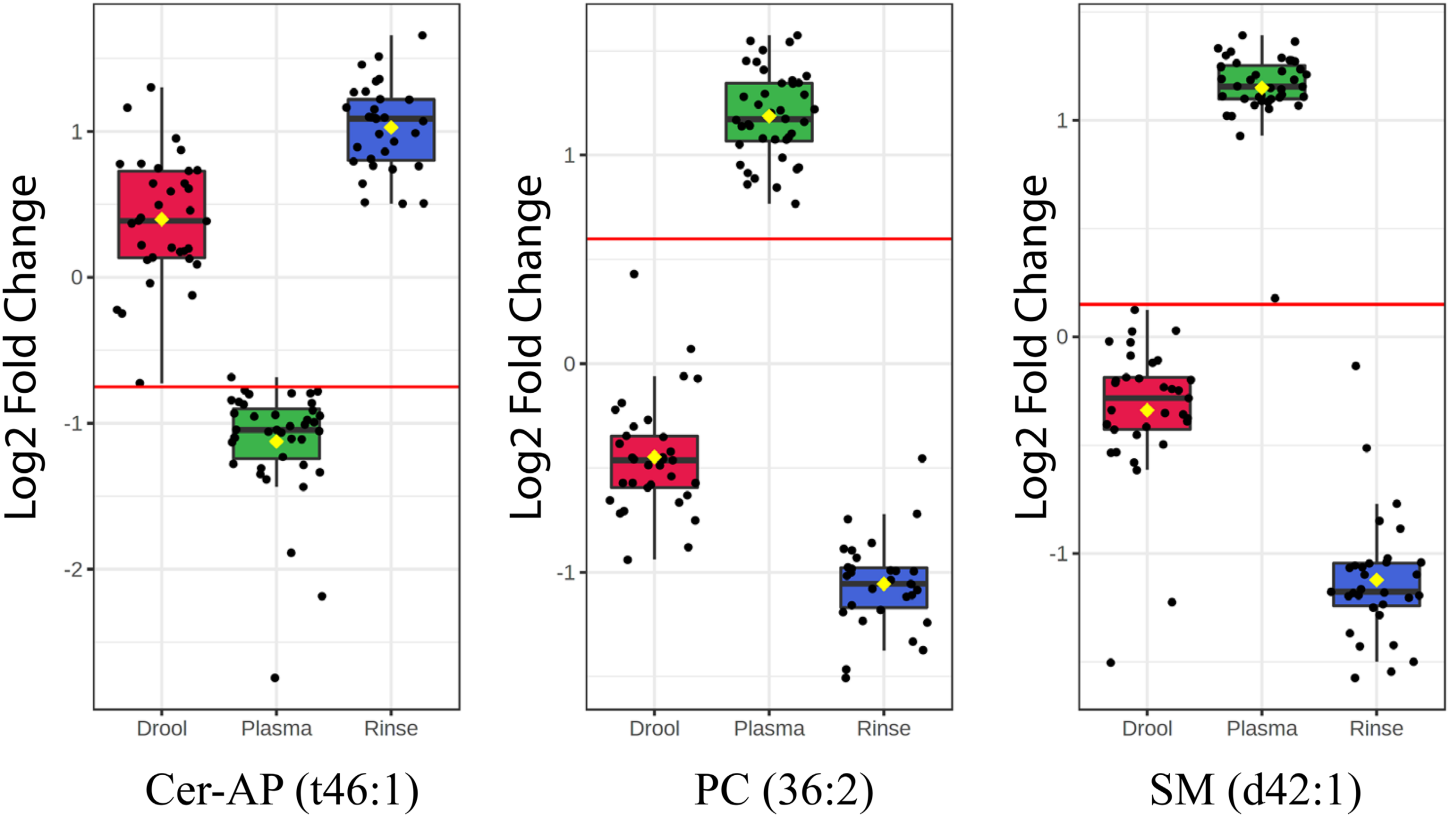
Key representatives of ceramide, phosphatidylcholine and sphingomyelin lipids demonstrate the behaviour of those lipid sub‐classes across UWMS, oral rinse and plasma of glioblastoma patients. Note: The horizontal red line indicates the threshold cut‐off for the true positive rate (sensitivity) of the metabolite within the 95% confidence interval. Cer, ceramides; PC, phosphatidylcholines; SM, sphingomyelins.

In contrast, the lipidome profile showed that the statistically significant lipids had a greater abundance in plasma compared to UWMS and rinse. The major lipid subgroups (AUROC = 1) observed across the samples consisted of ceramides, phosphatidylcholines and sphingomyelins. Of these, ceramides showed an elevation in saliva compared to plasma (Figure 3 and Figure S3).

### 3.3 Association of individual metabolites and lipidomic alterations with progression‐free survival of GBM patients

**FIGURE 4:**
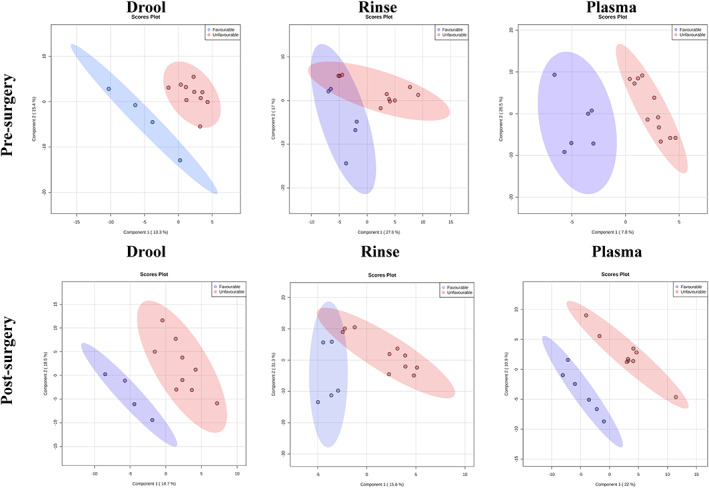
Patients with favourable outcomes (PFS ≥ 9 months) are represented in blue, and patients with unfavourable outcomes (PFS <9 months) in red. (A) Partial least squares‐discriminant analysis (PLS‐DA) for the metabolic profiles of UWMS, oral rinse samples and plasma samples of glioblastoma patients pre‐surgery. (B) Partial least squares‐discriminant analysis (PLS‐DA) for the metabolic profiles of UWMS, oral rinse and plasma samples of glioblastoma patients post‐surgery.

**FIGURE 5:**
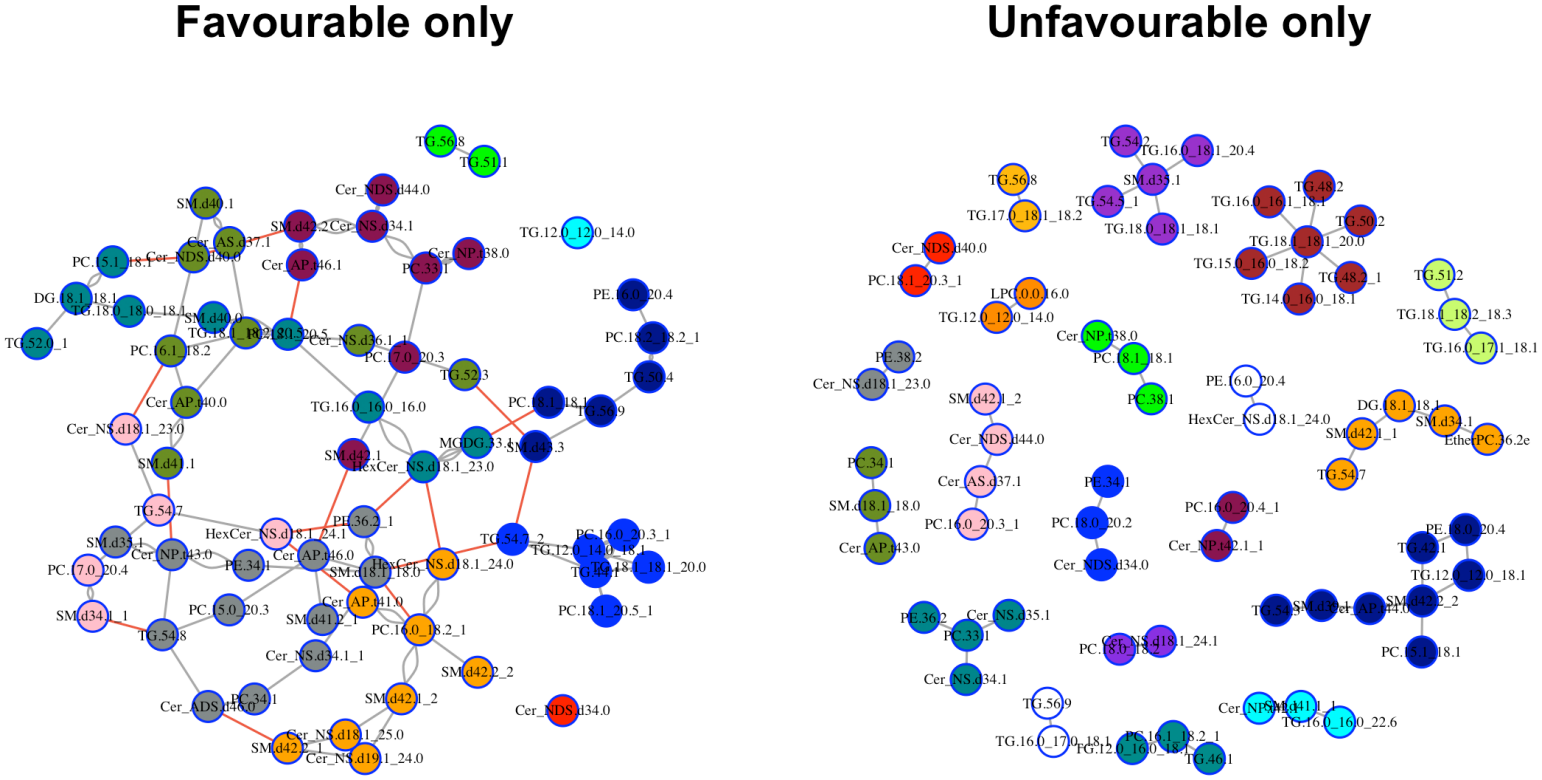
Graphical assessment using the Graphical LASSO. Lipids analysis across all body fluids of GBM patients with favourable outcomes (left panel) and unfavourable outcomes (right panel).

To evaluate the potential prognostic utility of alterations in the metabolome and lipidome of GBM patients, we divided the cohort between patients with unfavourable (PFS < 9 months) and favourable (PFS ≥ 9 months) outcomes. The summary of findings for pre and post‐surgery samples in saliva and plasma is shown in Table 1. Samples were considered statistically significant when meeting the *p* < 0.05 and a fold change >1.5 criteria. In the pathway analyses, altered metabolites shown in Table 1 were related to the valine, leucine and isoleucine biosynthesis in the pre‐surgery groups (*p* = 0.0047, Figure S4) and pentose phosphate pathway (*p* = 0.0343, Figure S5) in the post‐surgery group. In both pre and post‐surgery groups, partial least squares‐discriminant analysis (PLS‐DA) and volcano plots of up and downregulated metabolites for UWMS, rinse and plasma samples are shown in Figure 4 and Figure S6, respectively. When analysing the lipids identified in pre or post‐surgery samples, no lipid detected had *p* < 0.05 and a fold change >1.5, therefore, not reaching significant statistical alterations between both groups. For lipids, additional graphic network analyses using the graphical LASSO were performed (Figure 5). A more heterogeneous abundance of lipids, and fewer associations between them was found in patients with unfavourable outcomes in contrast to favourable outcomes. Patients who experienced favourable outcomes displayed a more homogeneous network with interlinked lipid clusters, whilst the patients with unfavourable outcomes showed fewer connections between markers, suggesting alterations in key lipids in the unfavourable state.

**TABLE 1:** Differentially abundant metabolites in patients with unfavourable outcomes compared to favourable outcomes across all samples.

	log2(FC)	raw.pval
Pre‐surgery – UWMS		
Indoline‐2‐carboxylate	−2.9953	0.0212
Cytosine	−1.8178	0.0333
2‐Ketobutyrate	−3.0748	0.0433
Adenosine 3‐5‐cyclic monophosphate	2.4282	0.0458
3‐Hydroxy‐dl‐kynurenine	3.3036	0.0483
Pre‐surgery – rinse		
l‐Dihydroorotic acid	6.9348	0.0391
dl‐Valine (D8)	−2.6159	0.0447
Pre‐surgery – plasma		
2‐4‐Quinolinediol	−2.1424	0.0004
Folinic acid	6.8334	0.0008
S‐2‐Aminoethyl‐l‐cysteine	0.87297	0.0318
4‐Hydroxy‐l‐glutamic acid	−5.4118	0.0341
Oxamic acid	−1.3963	0.0344
Galactonic acid	−2.3856	0.0409
Post‐surgery – UWMS		
4‐Hydroxybenzoic acid	−1.492	0.0022
Uridine 5‐diphosphate	7.2527	0.0022
L‐Arabinose	−2.373	0.0303
Post‐surgery – rinse		
O‐Phosphorylethanolamine	−6.3571	0.0231
Thymidine	−8.088	0.0349
beta‐Nicotinamide mononucleotide	9.4645	0.0379
Post‐surgery – plasma		
L‐Gluthathione (oxidised)	−3.6732	0.0042
Mevalonic acid 5‐phosphate	−3.9025	0.0069
Folinic acid	−1.7127	0.0155
4‐Quinolinol	−4.3469	0.0165
beta‐Nicotinamide mononucleotide	−2.1142	0.0191
5‐Methoxytryptamine	−5.4377	0.0203
D‐Fructose 1,6‐biphosphate	−2.2578	0.0251
cis‐Aconitic acid	2.602	0.0332
2‐3‐Dihydroxybenzoic acid	−1.7134	0.0344
d‐Xylulose‐5‐phosphate	−1.354	0.0371

Abbreviation: FC, fold change.

## 4 DISCUSSION

GBM remains the most common and fatal cancer in the central nervous system, despite advanced surgical resection methods and standard of care chemoradiation. While current treatment shows initial efficacy, tumours often recur and develop resistance to treatment. This is in part due to the invasive capacity of GBM, which contributes to the tumour's aggressiveness and resistance to treatment, culminating in high recurrence rates.^44^ Post‐treatment surveillance is currently done by MRI but even with optimal imaging, it is difficult to distinguish tumour recurrence from treatment effect.^3^ Therefore, investigating biomarkers to monitor disease progression and monitor treatment response in GBM patients is warranted. In this study, both saliva and blood were used as a source for the investigation of biomarkers. While blood has traditionally been the primary body fluid analysed in clinical settings, there has been a growing interest in using saliva as an alternative biofluid for sampling. This is due to the fact that saliva collection is simple, inexpensive and does not require specialised staff or equipment.

We have investigated metabolomics and lipidomics profiles as potential prognostic biomarkers in GBM. We have detected 13 altered metabolites between GBM patients with favourable and unfavourable outcomes across preoperative plasma and saliva samples, whereas 16 metabolites were identified in post‐operative samples. These metabolites are involved in regulating a number of pathways, including pentose phosphate pathway, valine, leucine and isoleucine biosynthesis. The amino acid metabolism is commonly upregulated in several cancer types, enabling the survival and growth of cancer cells.^45^

Overall, we have identified higher metabolite levels in saliva compared to plasma and more elevated lipids expression in plasma than in saliva. When comparing the results obtained between UWMS and oral rinse, we noticed some inconsistencies. This may be due to the fact that saliva is secreted from multiple glands within the oral cavity, therefore, salivary secretions from different glands have shown to have variable biochemical composition. While parotid gland secretion has been reported to have high content of proline‐rich proteins, sublingual gland secretion has elevated levels of glycoproteins. Sampling methods have shown that UWMS (sublingual gland secretion) produced higher levels of metabolites compared to secretions from other glands.

Across all samples tested, the glutamine and glutamate metabolism played a central role, particularly regarding the usual energy pathway of the citrate cycles. Similarly, several studies have suggested glutamine dependency by tumour cells.^46^, ^47^, ^48^ A considerable amount of glutamine/glutamate metabolism seemed geared primarily towards nucleotide biosynthesis, followed by the generation of arginine and proline metabolism intermediates and end‐products such as creatinine and citrulline (Figure 2). Recent studies in GBM cell lines indicated that enzymes such as glutamine synthase drive the glutamate → glutamine conversion as the first step towards de novo purine biosynthesis.^49^ Our observations support previous reports^49^ regarding an increase in purine levels caused by the glutamine input at the AICAR junction (Figure 2). Also, the glutamine/glutamate pathways were not geared towards glutaminolysis which is the usual route of energy pathways during cancer metabolism.

Arginine is an essential amino acid presenting key functions in various metabolic processes, such as the synthesis of proteins. When arginine is removed from the culture media, it leads cancer cell lines to death in a rapid way.^50^ Recently, it has been shown that increased citrulline levels reduce the effectivity of arginine deprivation therapy in GBM cells.^51^ This correlation is noteworthy since arginine deprivation therapy causes significant radio sensitisation in GBM cells.^51^, ^52^ Our results indicate that, when combined with the elevated purine levels, the increased arginine metabolism intermediates and byproducts might influence the clinical responses of patients.

Interestingly, we observed that creatinine and kynurenine were elevated in plasma of all patients when compared to saliva. Excess levels of these metabolites, caused by a disrupted tryptophan metabolism in the neuronal cells, have been shown to promote the kynurenine pathway, causing neurotoxicity and neuronal death.^53^ In addition, levels of metabolites in plasma, namely tyrosine, phenylalanine, glucose, creatine and creatinine presented a significant correlation with tumour grade.^27^ In contrast, greater abundance of ceramide lipid levels was observed in the saliva samples compared to plasma (Figure 3). An elevation in ceramide accumulation, primarily attributed to choline and tryptophan depletion, has been shown to trigger apoptotic pathways in PC12 cells^54^, ^55^ and suppress immune activity.^56^ It is also suggested that a ceramide accumulation triggered the ceramide → sphingomyelin conversion, and ceramide‐induced cellular apoptosis is avoided by this conversion.^57^, ^58^ The elevated sphingomyelin in GBM cells has also been shown in a recent lipidomic biomarker identification study.^59^

We found very few differentially abundant metabolites and lipids between GBM patients with favourable and unfavourable outcomes, most likely due to the small sample size. Similarly, Yu et al^31^ observed alterations in plasma metabolites in high‐grade glioma when compared to low‐grade or healthy controls. They found glycolytic metabolites to be increased in plasma samples, whilst the tricarboxylic acid cycle, citrate and succinate were decreased. In their study, Yu et al^31^ observed two increased metabolites (tyrosine and phenylalanine) in GBM patients compared to low‐grade glioma patients and healthy volunteers.

We found decreased levels of oxamic acid in plasma of GBM patients with unfavourable outcomes compared to favourable outcomes. Oxamic acid acts as an inhibitor of lactate dehydrogenase (LDH), which is normally increased in cancer cells. The overexpression of LDH correlates with poor prognoses in cancer patients.^60^, ^61^ In lipids, the samples from unfavourable groups appeared to have lost the association between markers when compared to patients with favourable outcomes. Other studies have already explored the differences in lipid metabolism to predict the risk of poor prognosis.^32^ In their research, prognosis‐related lipid metabolism genes were differentially expressed between GBM and low‐grade gliomas.^31^, ^32^ Similar to our findings, Zhou et al^30^ demonstrated a correlation network of lipids with more interactions in glioma patients with IDH mutation compared to wild‐type glioma patients. Mutations found in IDH affect cell metabolism, influencing patients' prognosis. Glioma patients with IDH1 and IDH2 mutations present a better outcome than patients with IDH wild‐type.^62^

The small sample size is a limitation of our study. We must analyse more patients to obtain precise markers and early indicators of GBM progression. In addition, in future studies, other groups, including healthy controls and non‐tumoural patients undergoing brain surgery, should be analysed. The power analysis of the current study indicated that a greater sample size (Figure S6) would improve the predictability of the high‐throughput (cumulative Q2 ≥ 0.9). The future analyses of larger cohorts can help enable the prediction of GBM progression using metabolites as biomarkers, helping in decision‐making in the GBM clinical setting.

## 5 CONCLUSION

Our findings suggest that metabolic alterations in plasma and saliva of GBM patients can be identified in a less invasive way and may be useful as prognostic biomarkers in the future. Further studies with larger cohorts need to be performed to validate our findings.

## AUTHOR CONTRIBUTIONS

**Juliana Muller Bark:** Conceptualization (equal); formal analysis (equal); writing – original draft (equal); writing – review and editing (equal). **Avinash Karpe:** Data curation (equal); formal analysis (equal); methodology (equal); writing – original draft (equal); writing – review and editing (equal). **James D Doecke:** Formal analysis (equal); writing – review and editing (equal). **Paul Leo:** Conceptualization (equal); writing – review and editing (equal). **Rosalind L. Jeffree:** Conceptualization (equal); writing – review and editing (equal). **Benjamin Chua:** Conceptualization (equal); writing – review and editing (equal). **Bryan W. Day:** Conceptualization (equal); writing – review and editing (equal). **David Beale:** Conceptualization (equal); formal analysis (equal); methodology (equal); writing – review and editing (equal). **Chamindie Punyadeera:** Conceptualization (equal); funding acquisition (equal); project administration (equal); writing – review and editing (equal).

## FUNDING INFORMATION

ATM LATAM QUT Postgraduate Research Scholarship funded JMB. RBWH Foundation projects grant funded this project. Cancer Australia (APP 1145657) and NHMRC Ideas Grants (APP 2002576 and APP 2012560), Royal Brisbane Women's Hospital Foundation and the Garnett Passé and Rodney Williams Foundation funded CP. B.W.D. is funded by the Sid Faithfull Group and Cure Brain Cancer Foundation.

## CONFLICT OF INTEREST STATEMENT

The authors declare no conflict of interest.

## ETHICS APPROVAL

Human research ethics approval was obtained by the committees of Royal Brisbane and Women's Hospital (Approval number: HREC/2019/QRBW/48780), Queensland University of Technology (Approval number: 1900000292), and The Griffith University Human Research Ethics Committee (GUHREC Ref No: 2022/061).

## Supporting information


Appendix S1.

Appendix S2.

## Data Availability

The data that supports the findings of this study are available in the supplementary material of this article.
