# Supplementary material for: A pilot study: Metabolic profiling of plasma and saliva samples from newly diagnosed glioblastoma patients
Source: Cancer Med. 2023 Apr 9;12(10):11427–37. doi: 10.1002/cam4.5857 (PMC10242862; doi:10.1002/cam4.5857)
Supplement: Supplementary file 1 — Appendix S1. [file CAM4-12-11427-s002.doc]

# **Metabolic profiling of plasma and saliva samples from newly diagnosed glioblastoma patients**

Juliana Müller Bark^1,4#^, Avinash V. Karpe^3#^, James Doecke^12^, Paul Leo^4,7^, Rosalind L. Jeffree^6,8,10,11^, Benjamin Chua^8,9^, Bryan W. Day^4,8,11^, David J. Beale^3^, Chamindie Punyadeera^2^*

Table S1. Participant’s information and clinical outcomes

| **#** | **Gender** | **Age** | **Date of collection / samples collected** | **Date of surgery** | **Date of f1 collection** | **Outcome** | **Date of outcome** | **Time elapsed since first collection** | **Considered as:** |
| --- | --- | --- | --- | --- | --- | --- | --- | --- | --- |
| **1** | M | 72 | 3/06/2019  Plasma, UWMS and Rinse | 5/06/2019 | 10/06/2019  Plasma, UWMS and Rinse | Deceased | 3/09/2019 | 0 Years, 3 Months, 0 Days | **Unfavourable** |
| **2** | M | 54 | 28/06/2019  Plasma, UWMS and Rinse | 1/07/2019 | 5/07/2019  Plasma, UWMS and Rinse | Deceased | 14/11/2019 | 0 Years, 4 Months, 17 Days | **Unfavourable** |
| **3** | M | 57 | 19/08/2019  Plasma and Rinse | 19/08/2019 | 22/08/2019  Plasma, UWMS and Rinse | Progression | 24/04/2021 | 1 Years, 8 Months, 5 Days | **Favourable** |
| **4** | F | 69 | 19/08/2019  Plasma, UWMS and Rinse | 20/08/2019 | 23/08/2019  Plasma and Rinse | Progression (09/12/2020) + Progression (31/03/21) | 9/12/2020 | 1 Years, 3 Months, 20 Days | **Favourable** |
| **5** | M | 61 | 11/09/2019  Plasma, UWMS and Rinse | 11/09/2019 | 20/09/2019  Plasma, UWMS and Rinse | 1st Progression, progression on the 17/06/21, deceased on the 31/08/21 | 17/08/2020 | 0 Years, 11 Months, 6 Days | **Favourable** |
| **6** | F | 65 | 16/09/2019  Plasma, UWMS and Rinse | 17/09/2019 | 19/09/2019  Plasma, UWMS and Rinse | Likely progression - 17/05/20) + Deceased 28/08/2020 = 11 months after collection | 17/05/2020 | 0 Years, 8 Months, 1 Days | **Unfavourable** |
| **8** | F | 51 | 6/01/2020  Plasma, UWMS and Rinse | 6/01/2020 | 9/01/2020  Plasma, UWMS and Rinse | Progression | 17/09/2020 | 0 Years, 8 Months, 11 Days | **Unfavourable** |
| **9** | F | 52 | 6/01/2020  UWMS and Rinse | 7/01/2020 | 9/01/2020  Plasma and Rinse | Deceased | 25/09/2020 | 0 Years, 8 Months, 19 Days | **Unfavourable** |
| **10** | M | 43 | 13/01/2020  Plasma, UWMS and Rinse | 16/01/2020 | 23/01/2020  Plasma, UWMS and Rinse | Deceased | 25/02/2020 | 0 Years, 1 Months, 12 Days | **Unfavourable** |
| **11** | F | 82 | 23/01/2020  Plasma, UWMS and Rinse | 24/01/2020 | 30/01/2020  Plasma, UWMS and Rinse | Deceased | 8/05/2020 | 0 Years, 3 Months, 15 Days | **Unfavourable** |
| **12** | F | 68 | 26/06/2020  Plasma | 26/06/2020 | 1/07/2020  Plasma | Unknown, no MRI since 8/2020. Nill treatment with declining functions. | 1/03/2021 | 0 Years, 8 Months, 3 Days | **Unknown** |
| **13** | F | 37 | 23/07/2020  Plasma, UWMS and Rinse | 23/07/2020 | 29/07/2020  Plasma, UWMS and Rinse | Progression | 29/01/2021 | 0 Years, 6 Months, 6 Days | **Unfavourable** |
| **14** | F | 67 | 23/07/2020  Plasma, UWMS and Rinse | 24/07/2020 | 29/07/2020  Plasma, UWMS and Rinse | Progression | 17/03/2021 | 0 Years, 7 Months, 22 Days | **Unfavourable** |
| **15** | F | 64 | 28/07/2020  Plasma, UWMS and Rinse | 29/07/2020 | 3/08/2020  Plasma, UWMS and Rinse | Progression 12/05/21. stable disease on the 28/01/2021. deceased on the 11/07/21 | 12/05/2021 | 0 Years, 9 Months, 14 Days | **Favourable** |
| **16** | M | 70 | 25/08/2020  Plasma | 25/08/2020 | 27/08/2020  Plasma | Deceased | 15/12/2020 | 0 Years, 3 Months, 20 Days | **Unfavourable** |
| **17** | M | 69 | 11/09/2020  Plasma, UWMS and Rinse | 14/09/2020 | 18/09/2020  Plasma, UWMS and Rinse | Unknown (16/07/2021)No progression after 4 months of collection (11/09/2020). progression on the 24/07/21. deceased on the 31/07/2021 | 24/07/2021 | 0 Years, 10 Months, 13 Days | **Favourable** |
| **18** | M | 60 | 25/09/2020  Plasma, UWMS and Rinse | 25/09/2020 | 28/09/2020  UWMS and Rinse | Deceased | 5/01/2021 | 0 Years, 3 Months, 11 Days | **Unfavourable** |
| **19** | F | 67 | 7/10/2020  Plasma, UWMS and Rinse | 7/10/2020 | 9/10/2020  Plasma, UWMS and Rinse | Progression on the 18/02/2021. relapse on the 22/04/2021. deceased on the 22/05/2021 | 18/02/2021 | 0 Years, 4 Months, 11 Days | **Unfavourable** |
| **20** | M | 58 | 4/11/2020  Plasma, UWMS and Rinse | 4/11/2020 | 9/11/2020  Plasma, UWMS and Rinse | Unkown on 5/07/2021. progression on the 11/08/2021. deceased on the 04/10/2021 | 11/08/2021 | 0 Years, 9 Months, 7 Days | **Favourable** |
| **21** | F | 47 | 13/11/2020  Plasma, UWMS and Rinse | 13/11/2020 | 18/11/2020  Plasma, UWMS and Rinse | No progression | 7/06/2021 | 0 Years, 6 Months, 25 Days | **Unkown** |
| **22** | F | 64 | 13/01/2021  Plasma | 13/01/2021 | 18/01/2021  Plasma | Unkown, pseudoprogression and radiation necrosis could both have this appearance (MRI 17/7/21) | 15/07/2021 | 0 Years, 6 Months, 2 Days | **Unkown** |

Table S2. Variability among the quality control metabolites applied for the LC-MS analysis.

| **Metabolite** | **Relative Standard Deviation (%)** |
| --- | --- |
| L-Arginine | 9.51 |
| L-Histidine | 9.10 |
| L-Cystine | 12.74 |
| L-Serine | 6.44 |
| L-Threonine | 7.63 |
| L-Proline | 8.12 |
| L-Methionine | 7.92 |
| L-Tyrosine | 10.99 |
| L-Phenylalanine | 5.36 |
| L-Glutamic acid | 9.93 |
| L-Aspartic Acid | 14.87 |
| L-Tryptophan | 5.25 |
| Succinic acid | 6.86 |
| L-Malic acid | 1.31 |
| Citric acid | 2.54 |

**Figure S1**. OPLS-DA dataset for the metabolic profile of various samples derived from glioblastoma patients. The figures indicate (A) Spread of samples indicated by score scatter plot, with ellipse representing 95% confidence interval, and (B) Spread of metabolites with respect to the groups, indicated by the loading scatter plot. N =125, R^2^X = 0.805; R^2^Y = 0.895; R^2^= 0.93.


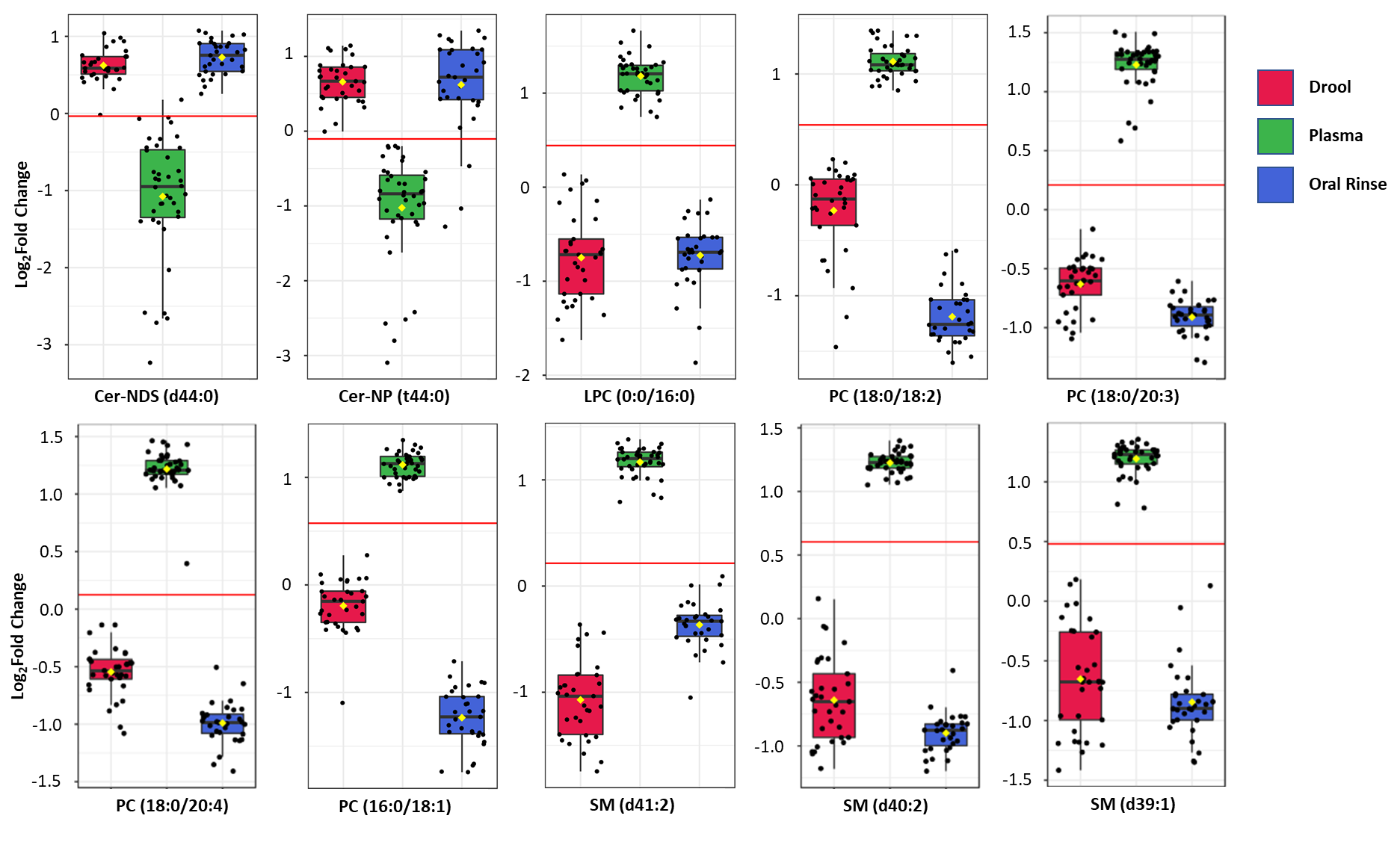


**Figure S2**. Ceramide, phosphotidylcholine and sphingomycelin lipids demonstrating the behaviour of those lipid sub-classes during glioblastoma across UWMS, plasma and oral rinse of glioblastoma patients. Note: The horizontal red line indicates the threshold cut-off for the true positive rate (sensitivity) of the metabolite within the 95% confidence interval.


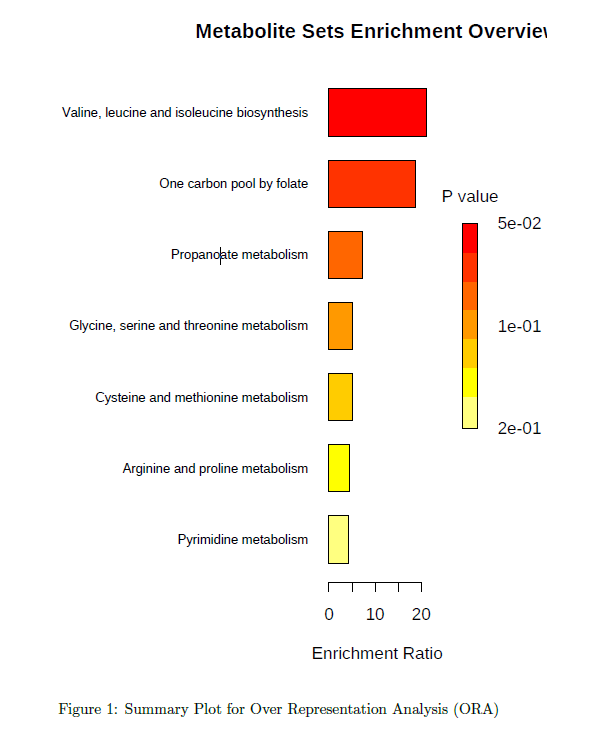


**Figure S3.** Summary plot of the pathway analyses of metabolites from table 2 in the pre-surgery groups.


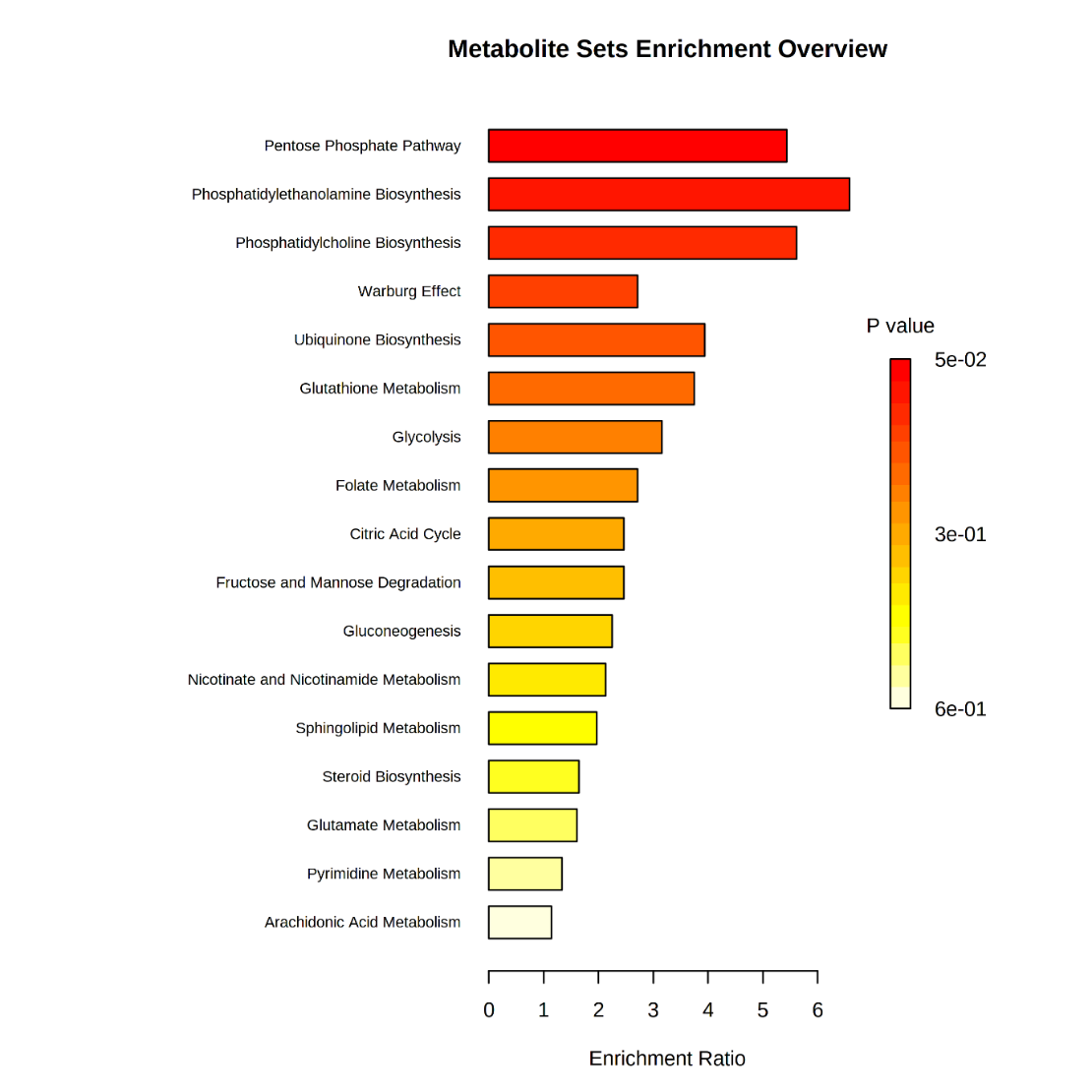


**Figure S4.** Summary plot of the pathway analyses of metabolites from table 2 in the post-surgery groups.


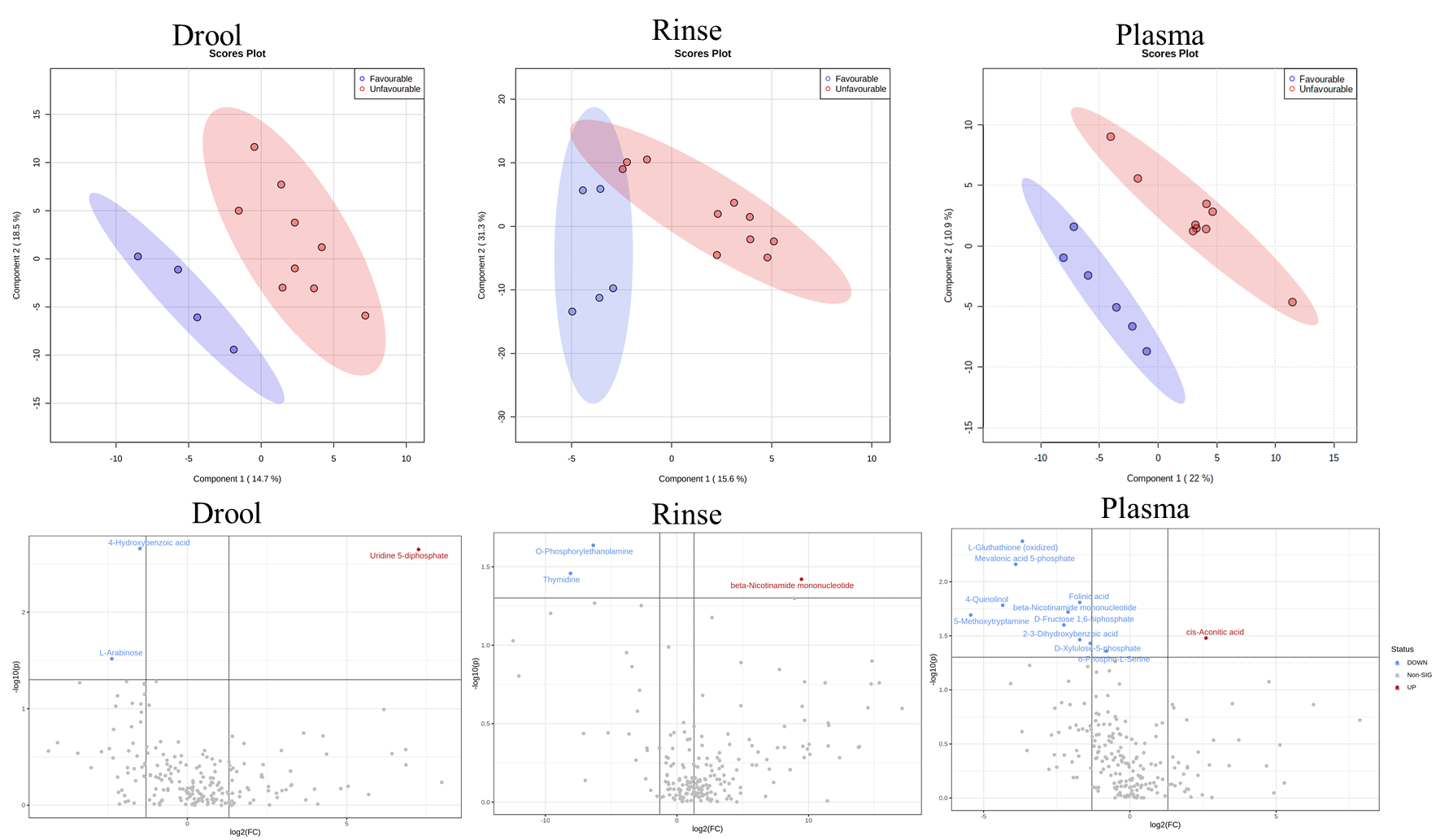

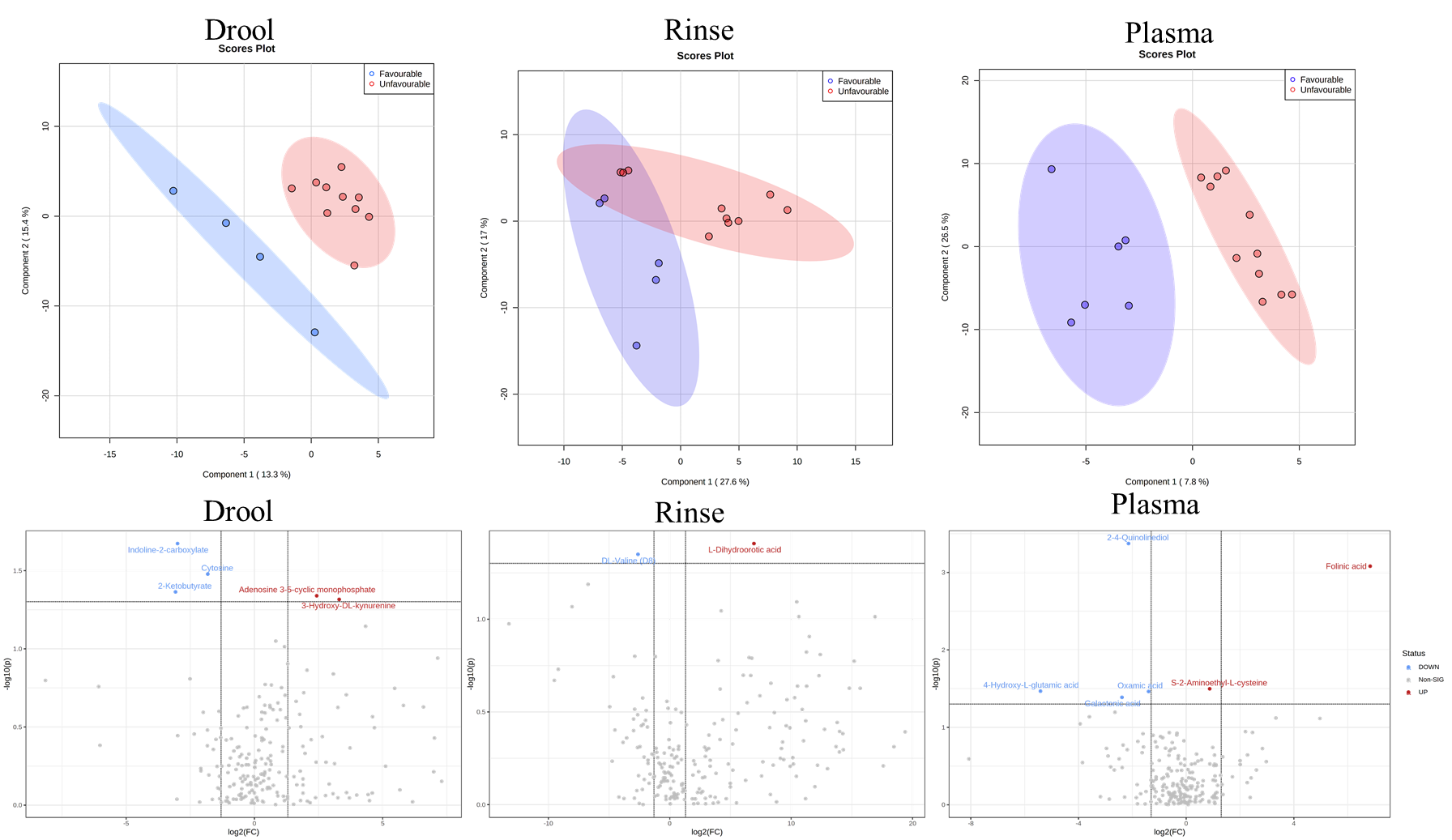


**Figure S5.** A) Volcano plot of all metabolites identified in UWMS, rinse and plasma of glioblastoma patients pre-surgery. Red dots represent upregulated metabolites, while blue dots correspond to downregulates metabolites (p‐values <0.05 and a fold change >1.5). Grey dots are metabolites with no statistical difference. B) Volcano plot of all metabolites identified in UWMS, rinse and plasma of glioblastoma patients post-surgery. Red dots represent upregulated metabolites, while blue dots correspond to downregulates metabolites (p‐values < 0.05 and a fold change > 1.5). Grey dots are metabolites with no statistical difference.


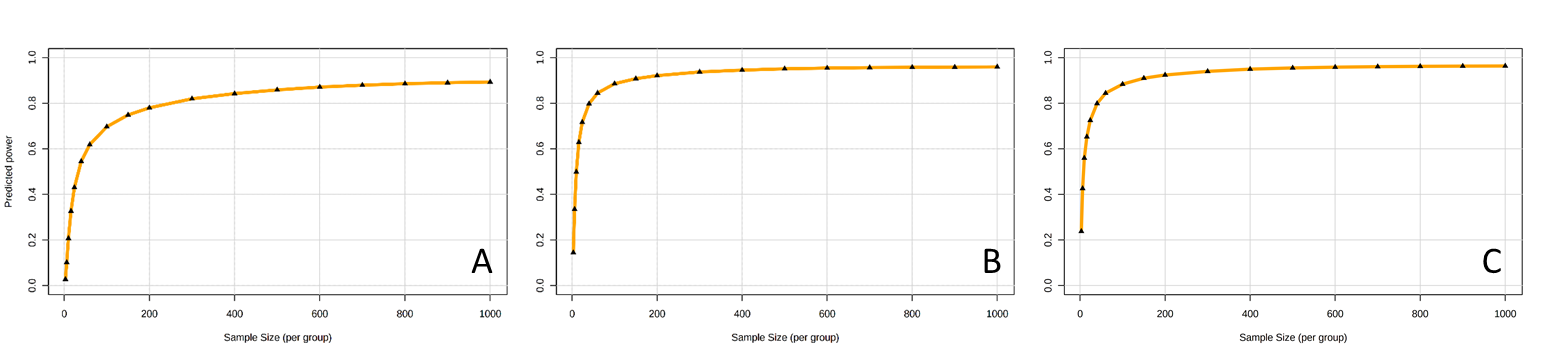


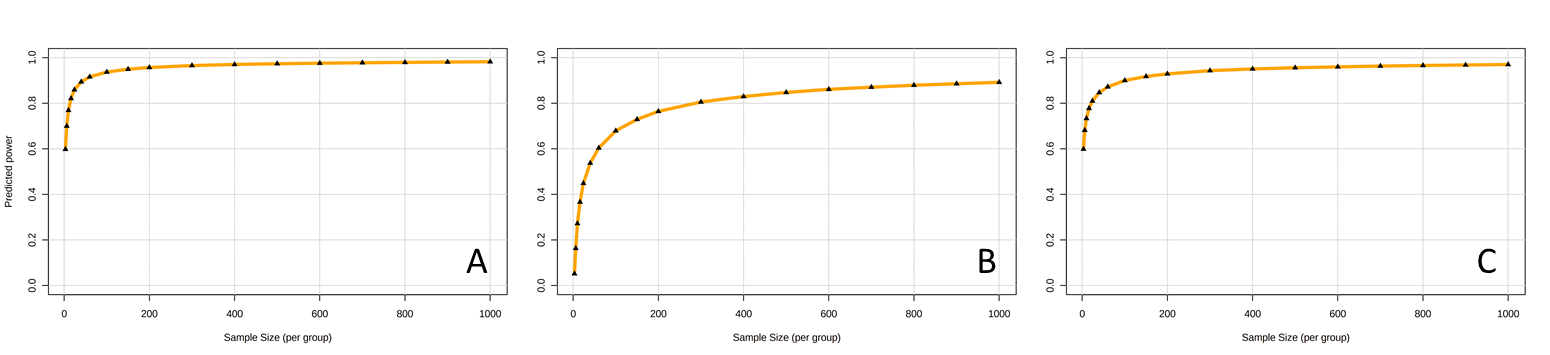
**Figure S6. Power analysis *Upper panel.*** Power analysis results of the metabolic profile within (A) UWMS vs Plasma (B) UWMS vs Rinse and (C) Plasma vs Rinse matrices for predicting the sample size to obtain a false discovery rate of 0.05. ***Bottom panel***. Power analysis results of the lipidomic profile within (A) UWMS vs Plasma (B) UWMS vs Rinse and (C) Plasma vs Rinse matrices for predicting the sample size to obtain a false discovery rate of 0.05.
